# Lower Rates of Hypocalcemia Following Near-Infrared Autofluorescence Use in Thyroidectomy: A Meta-Analysis of RCTs

**DOI:** 10.3390/diagnostics14050505

**Published:** 2024-02-27

**Authors:** Karthik N. Rao, Renu Rajguru, Prajwal Dange, Diana Vetter, Frederic Triponez, Iain J. Nixon, Gregory W. Randolph, Antti A. Mäkitie, Mark Zafereo, Alfio Ferlito

**Affiliations:** 1 Department of Head and Neck Oncology, All India Institute of Medical Sciences, Raipur 492099, India; prajwal.dange@gmail.com; 2 Sri Shankara Cancer Hospital and Research Center, Bangalore 560004, India; 3 Department of Otorhinolaryngology and Head Neck Surgery, All India Institute of Medical Sciences, Raipur 492099, India; renurajguru@yahoo.com; 4 Department of Visceral and Transplant Surgery, University Hospital Zurich, 8032 Zurich, Switzerland; diana.vetter@usz.ch; 5 Department of Thoracic and Endocrine Surgery, University Hospitals of Geneva, 1205 Geneva, Switzerland; frederic.triponez@hcuge.ch; 6 Department of Surgery and Otolaryngology, Head and Neck Surgery, Edinburgh University, Edinburgh EH3 9YL, UK; iain.nixon@nhslothian.scot.nhs.uk; 7 Division of Thyroid and Parathyroid Endocrine Surgery, Department of Otolaryngology-Head and Neck Surgery, Massachusetts Eye and Ear Infirmary, Harvard Medical School, Boston, MA 02114, USA; gregory_randolph@meei.harvard.edu; 8 Department of Surgery, Massachusetts General Hospital, Harvard Medical School, Boston, MA 02114, USA; 9 Department of Otorhinolaryngology, Head and Neck Surgery, Research Program in Systems Oncology, Faculty of Medicine, University of Helsinki, Helsinki University Hospital, 00014 Helsinki, Finland; antti.makitie@hus.fi; 10 Department of Head & Neck Surgery, MD Anderson Cancer Center, Houston, TX 77005, USA; mzafereo@mdanderson.org; 11 Coordinator of the International Head and Neck Scientific Group, 35100 Padua, Italy; profalfioferlito@gmail.com

**Keywords:** thyroid gland, total thyroidectomy, hypocalcemia, near-infrared autofluorescence, outcomes

## Abstract

Background: Iatrogenic injury of the parathyroid glands is the most frequent complication after total thyroidectomy. Objective: To determine the effectiveness of near-infrared autofluorescence (NIRAF) in reducing postoperative hypocalcemia following total thyroidectomy. Methods: PubMed, Scopus, and Google Scholar databases were searched. Randomised trials reporting at least one hypocalcemia outcome following total thyroidectomy using NIRAF were included. Results: The qualitative data synthesis comprised 1363 patients from nine randomised studies, NIRAF arm = 636 cases and non-NIRAF arm = 637 cases. There was a statistically significant difference in the overall rate of hypocalcemia log(OR) = −0.7 [(−1.01, −0.40), M-H, REM, CI = 95%] and temporary hypocalcemia log(OR) = −0.8 [(−1.01, −0.59), M-H, REM, CI = 95%] favouring the NIRAF. The difference in the rate of permanent hypocalcemia log(OR) = −1.09 [(−2.34, 0.17), M-H, REM, CI = 95%] between the two arms was lower in the NIRAF arm but was not statistically significant. Conclusions: NIRAF during total thyroidectomy helps in reducing postoperative hypocalcemia. Level of evidence—1.

## 1. Introduction

Iatrogenic injury of the parathyroid glands is the most frequent complication after total thyroidectomy and can result in hypoparathyroidism [1]. The reported incidence of hypocalcemia following total thyroidectomy exhibits a wide range. Temporary hypoparathyroidism is estimated to occur in 19–38% of cases, while permanent hypoparathyroidism occurs in 0–3% after total thyroidectomy [2]. Damage to the parathyroid glands can result in hypoparathyroidism, leading to hypocalcemia and long-term metabolic problems [3]. Hypocalcemia can increase morbidity, including cardiac arrhythmias and tetany, resulting in prolonged hospitalisation and potential mortality. Intraoperative guidance to identify and assess the parathyroid glands during thyroid surgery may help prevent surgical damage and improve postoperative outcomes and quality of life. Traditional methods to reduce the risk of surgical hypoparathyroidism have primarily relied on the ability of the surgeon to identify and carefully preserve the parathyroids and their vascular pedicles. However, intraoperative identification of the parathyroid glands can be difficult because of their variable position, their relatively small size, and their similarity to thyroid tissue, lymph nodes, or brown fat [4].

Intraoperative near-infrared autofluorescence (NIRAF) has emerged as a technique for identifying the parathyroid glands, with several studies demonstrating its effectiveness [5]. When the parathyroid glands are exposed to near-infrared excitation light at a wavelength of 785 nm [6], their intrinsic fluorophore, which is as of yet still unidentified, emits light in the near-infrared spectrum (700–900 nm) [7]. This emitted light can be detected by camera-based and probe-based systems providing real-time imaging for surgical guidance. Recent years have seen multiple randomised controlled trials investigating the use of NIRAF during total thyroidectomy to assess its potential in reducing the incidence of postoperative hypocalcemia and facilitating parathyroid gland identification and evaluation [8,9]. 

The European Society of Endocrinology clinical guideline defines hypoparathyroidism as having hypocalcemia (ionic Ca^2+^ < 4.61 mg/dL), abnormally low parathormone (PTH) levels, or requirement for ongoing active vitamin D therapy [10]. It is considered permanent if a condition persists for six months or more or necessitates continual calcium and active vitamin D therapy.

Our study represents the first meta-analysis including randomised controlled trials solely on the use of NIRAF during thyroidectomy to reduce hypocalcemia, aiming to determine the effectiveness of NIRAF in reducing postoperative hypocalcemia following total thyroidectomy.

## 2. Methodology

### 2.1. Reporting and Registration

This manuscript has strictly adhered to the PRISMA (preferred reporting items for systematic reviews and meta-analyses) [11] and AMSTAR (assessing the methodological quality of systematic reviews) guidelines [12]. The meta-analysis underwent a thorough evaluation by AMSTAR and was determined to be of high quality. This meta-analysis was prospectively registered in the international prospective register of systematic reviews (PROSPERO) CRD42023434610 [13]. An ethical declaration is not relevant as this investigation relies solely on published literature.

### 2.2. Search Strategy

We incorporated PubMed, Scopus, and Google Scholar repositories to ensure thorough inclusion of published literature. All published literature in English between 2010 to 2023 was considered.

### 2.3. Search Query Structure

“Parathyroid glands”, “Total thyroidectomy”, “Autofluorescence”, “Hypoparathyroidism”, “Hypocalcemia”, “Near infrared autofluorescence”, “Postoperative”, “Complications”, “Outcomes”. Logical operators (NOT, AND, OR) were employed to acquire the outcomes. The data was last retrieved on 10 June 2023.

### 2.4. Data Screening and Selection

**Figure 1:**
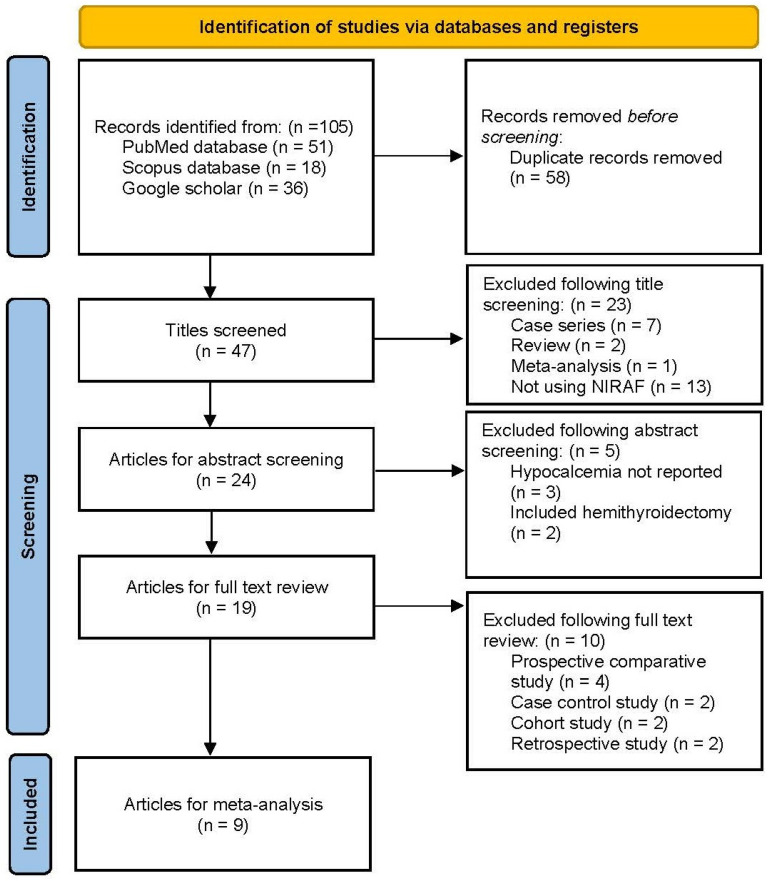
PRISMA flow diagram.

Two researchers (KNR and PD) independently screened the retrieved papers, considering the article type, title, and abstract. The qualified publications were aggregated, and an in-depth analysis of the full texts, along with references in pertinent articles, was conducted through snowball searching for similar studies (Figure 1). The article selection process was carried out by KNR and PD, with any disputes regarding inclusion resolved by the senior author, RR.

### 2.5. Inclusion and Exclusion Criteria

The inclusion criteria were treatment-naïve benign or malignant thyroid lesions undergoing total thyroidectomy with or without neck dissection and using NIRAF with or without indocyanine green (ICG) angiography for parathyroid identification during total thyroidectomy by experienced surgeons. Only randomised controlled trials reporting either overall, temporary, or permanent hypocalcemia, published in peer-reviewed journals, were included. 

The exclusion criteria were non-human studies and studies including patients with hemithyroidectomy, completion thyroidectomy, revision thyroid surgery, previous oncological treatment, and patients with second or recurrent cancer. Review articles, abstracts from meetings, case series and reports, letters to editor, retrospective series, non-randomised prospective studies were excluded.

### 2.6. Data Retrieval

KNR and PD independently screened all included articles. The recorded study characteristics encompassed the author, publication year, country, sample size, study type, level of evidence, Newcastle–Ottawa scale (NOS), risk of bias, total thyroidectomy cases, cases in the NIRAF arm, cases in the non-NIRAF arm, overall hypocalcemia rate, temporary hypocalcemia rate, permanent hypocalcemia rate, malignant and benign cases in the cohort, NIRAF assessment type, equipment type, parathyroid identification rate, accidental parathyroidectomy, parathyroid auto-transplantation rate, hypocalcemia definition in the study, and mean postoperative day 1 (POD 1) PTH levels (Table 1).

## 3. Quality Assessment

### 3.1. Level of Evidence

KNR and PD independently assessed the level of evidence in the eligible studies using the Oxford Centre for Evidence-Based Medicine (OCEBM).

### 3.2. Methodology Quality

The methodological quality was evaluated by two authors (KNR and PD) using the NOS [21]. Scores ranged from zero to nine, and articles with a score exceeding five were chosen for inclusion in the meta-analysis.

### 3.3. Risk of Bias Assessment

To assess bias, the Cochrane randomised trial tool (RoB2) was used [22]. We evaluated the following domains: random sequence generation (selection bias), allocation concealment (selection bias), incomplete outcome data (attrition bias), selective reporting (reporting bias), and other relevant domains. The studies were categorized as low risk, unclear risk, or high risk using RevMan v.5.4 (Cochrane Collaboration, Copenhagen, Denmark) [23] (Supplementary Figures S1 and S2).

### 3.4. Statistical Analysis

*Outcome Measure Analysis*: The assessment employed the log odds ratio (logOR) to examine differences in outcome measures [24]. Utilizing a random-effects model, we considered both within-study and between-study variability, allowing for fluctuations in the actual effect size across studies—a more conservative approach than a fixed-effects model [25].

*Visualization of Effect Size*: Forest and L’abbe plots were utilized for visualizing effect size estimates [26].

*Heterogeneity Testing*: To assess heterogeneity, the DerSimonian–Laird estimator determined the between-study variance (tau^2^), reflecting variations in true effect sizes among studies. The Cochran Q-test examined significant heterogeneity in true outcomes based on individual study effect sizes compared to the overall effect size [27].

*Higgins Test Interpretation*: Higgins I^2^, expressed as a percentage, indicated the proportion of total variability attributed to heterogeneity. A 0% value denoted no observed heterogeneity, while higher values signified increased heterogeneity. In cases of detected heterogeneity (tau^2^ > 0), a prediction interval was provided to accommodate uncertainty arising from heterogeneity [28].

*Identification of Outliers*: Outliers were determined through studentized residuals, considering the variability of estimated effect sizes and their standard errors [29]. Studies exceeding a specific threshold (calculated with the Bonferroni correction) were considered potential outliers, visualized in a radial plot [30].

*Assessment of Influential Studies*: Cook’s distances were employed to identify influential studies, quantifying each study’s impact on the overall model. Studies exceeding a specific threshold (median plus six times the interquartile range of Cook’s distances) were deemed influential [31].

*Publication Bias Analysis*: Additionally, funnel plot asymmetry was assessed to detect potential publication bias, where smaller studies with non-significant results might be underrepresented. Rank correlation and regression tests, utilizing the standard error of observed outcomes as the predictor, were applied to check for funnel plot asymmetry [32].

*Software Utilization*: All computations were carried out using the R Project for Statistical Computing v4.3.1 for Windows [33]. 

## 4. Results

### 4.1. Literature Retrieval and Data Extraction

The initial literature search using search syntax identified 105 articles (Figure 1). Of these, 47 remained after deleting 58 duplicates. Upon title screening, 23 articles were removed due to nonconformity with our study (case series = 7, review article = 2, meta-analysis = 1, not using NIRAF = 13). Following the abstract screening, five more articles were excluded (hypocalcemia not reported = 3, including hemithyroidectomy in the study = 2). After a full-text analysis of the remaining 19 articles, 10 papers were rejected due to being a prospective comparative study (n = 4), case-control study (n = 2), cohort study (n = 2), and retrospective study (n = 2). Finally, nine randomised controlled studies comparing NIRAF with non-NIRAF use in thyroidectomy [8,9,14,15,16,17,18,19,20] were included for meta-analysis. 

### 4.2. Quality of Included Studies

The main characteristics of the included studies are summarised in Table 1 and Table 2. The qualitative data synthesis yielded 1363 cases undergoing total thyroidectomy from nine articles. A total of 636 cases were randomised to the NIRAF arm, and 637 were randomised into the non-NIRAF arm. Seven studies reported the overall rates of postoperative hypocalcemia; all reported the rates of temporary postoperative hypocalcemia, and only six reported permanent hypocalcemia. Six studies were from Europe (France = 1, Greece = 1, Ukraine = 1, Germany = 1, Denmark = 1, and Italy = 1), two were from China, and one from Argentina (Table 2). Based on the risk of bias assessment tool, the included studies had a high risk of random sequence generation and allocation concealment (selection bias) and incomplete outcome reporting (attrition bias, selective reporting, or reporting bias). (Supplementary Figure S1).

### 4.3. Publication Bias

The funnel plot appears symmetric for the domains in the meta-analysis, suggesting a very low risk of publication bias among the included articles (Supplementary Figure S2).

### 4.4. The Overall Rate of Hypocalcemia

**Figure 2:**
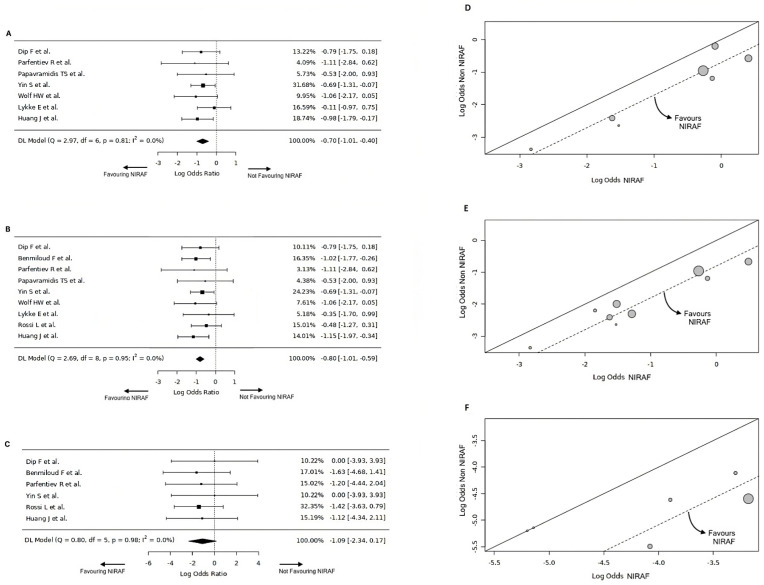
(**A**) Forest plot for overall rate of postoperative hypocalcemia [9,14,16,17,18,19,20]. (**B**) Forest plot for the rate of temporary hypocalcemia [8,9,14,15,16,17,18,19,20]. (**C**) Forest plot for the rate of permanent hypocalcemia [8,9,14,15,16,20]. (**D**) L’abbe plot for overall rate of postoperative hypocalcemia. (**E**) L’abbe plot for the rate of temporary hypocalcemia. (**F**) L’abbe plot for the rate of permanent hypocalcemia.

The overall hypocalcemia (OH) rate data were extracted from seven studies [9,14,16,17,18,19,20] for NIRAF (n = 415) and non-NIRAF (n = 417) arms. The pooled OH was 12.6% for the NIRAF arm and 20.1% for the non-NIRAF arm. The pooled data had no significant heterogeneity with Cochran Q = 2.97, Tau^2^ = 0, df = 6 (*p* = 0.81), or I^2^ = 0%. The log(OR) was −0.7 [(−1.01, −0.40), M-H, REM, CI = 95%], favouring the NIRAF arm. The overall effect estimates for the NIRAF and non-NIRAF arms were statistically significant for OH (Z = −3.95, *p* < 0.001, CI = 95%) (Figure 2A,D). Table 3 shows an overview of the meta-analysis statistics.

### 4.5. Temporary Hypocalcemia

The temporary hypocalcemia (TH) rate data were extracted from the nine NIRAF (n = 636) and non-NIRAF arm (n = 637) studies [8,9,14,15,16,17,18,19,20]. The pooled OH was 13.8% for the NIRAF arm and 24.8% for the non-NIRAF arm. The pooled data had no significant heterogeneity with Cochran Q = 2.69, Tau^2^ = 0, df = 8 (*p* = 0.95), or I^2^ = 0%. The log(OR) was −0.8 [(−1.01, −0.59), M-H, REM, CI = 95%], favouring the NIRAF arm. The overall effect estimates for the NIRAF and non-NIRAF arms were statistically significant for TH (Z = −5.14, *p* < 0.001, CI = 95%) (Figure 2B,E).

### 4.6. Permanent Hypocalcemia

The permanent hypocalcemia (PH) rate data were extracted from the six NIRAF (n = 476) and non-NIRAF arms (n = 473) studies [8,9,14,15,16,20]. The pooled PH was 0.2% for the NIRAF arm and 1.3% for the non-NIRAF arm. The pooled data had no significant heterogeneity with Cochran Q = 0.8, Tau^2^ = 0, df = 3 (*p* = 0.98), or I^2^ = 0%. The log(OR) was −1.09 [(−2.34, 0.17), M-H, REM, CI = 95%] was favouring NIRAF arm. The overall effect estimates for NIRAF and non-NIRAF arms were not statistically significant for PH (Z = −1.69, *p* = 0.09, CI = 95%) (Figure 2C,F).

### 4.7. Citation Network

**Figure 3:**
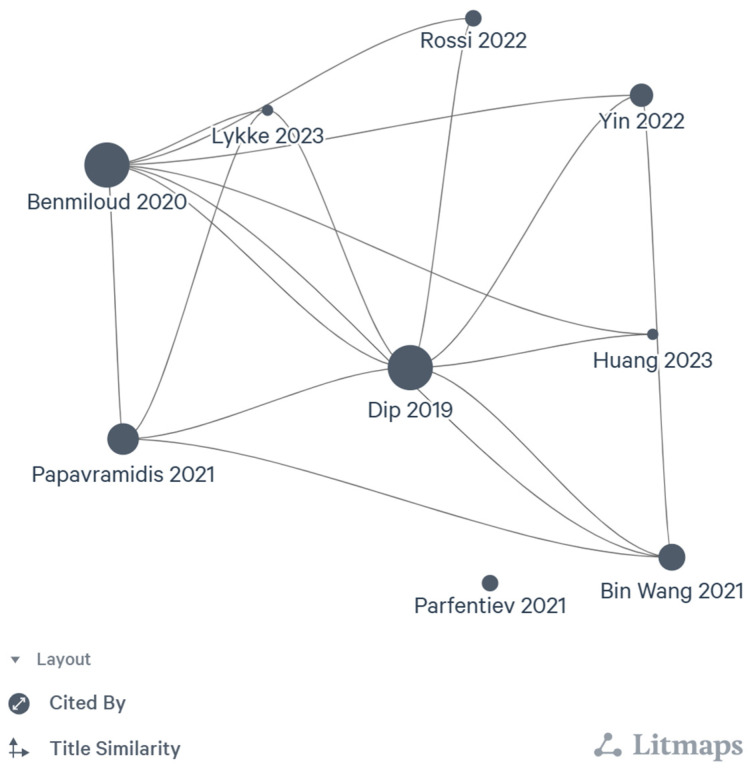
Citation network [8,9,14,15,16,17,18,19,20].

The citation network of the included articles was generated using the Litmaps tool. The axes on the literature citation maps are the logarithmic scale of citations and are distributed over the positions of articles according to the semantic similarity of their titles. The size of the individual article bubble corresponds to the logarithmic scale of the article citation. It shows that the early randomised NIRAF trials had more citation networks than others. Nearly all included studies have quoted Dip et al. [14], followed by Benmiloud et al. [15]. Parfentiev et al. [16] is the only study that does not cite any of the included studies (Figure 3).

## 5. Discussion

This study is the first attempt to systematically evaluate and summarise data from high-quality, non-heterogenous, randomised controlled trials on postoperative hypocalcemia after total thyroidectomy using NIRAF. We hope this will inform and guide the introduction of this new NIRAF technology.

NIRAF imaging has evolved as a non-invasive technique since its initial description by Paras et al. [34], who identified the potential to detect fluorescence emissions from parathyroid glands using near-infrared light. NIRAF imaging can be performed at any stage of the surgical procedure without significant interference or time commitment. It allows for autofluorescence detection in excised and intact parathyroid glands, enabling surgeons to salvage inadvertently removed glands before pathological analysis and perform auto-implantation. All of the included randomised controlled trials investigating the role of NIRAF in total thyroidectomy have demonstrated their significant impact on improving detection rates and reducing the occurrence of overall and temporary hypocalcemia [8,9,14,15,16,17,18,19,20].

Benmiloud et al. [15] conducted a study in France that included cases of total thyroidectomy with or without neck dissection performed by experienced surgeons. Their cohort exhibited an overall malignancy rate of 23.2%, with 76.8% of patients being benign. The study focused on NIRAF assessment without ICG angiography, utilising Fluobeam 800 (Fluoptics, Grenoble, France) for evaluation. Notably, there was a statistically significant difference in the identification of all four parathyroid glands in the NIRAF arm [47%] compared to the non-NIRAF arm (19.2%) (*p* < 0.001). Additionally, the rate of accidental parathyroidectomy in the NIRAF arm (2.4%) was significantly lower than in the non-NIRAF arm (11.5%) (*p* = 0.006). The study defined hypocalcemia as corrected calcium levels < 8 mg/dL. Interestingly, the mean PTH levels on POD 1 were 33.2 pg/mL in the NIRAF arm and 28.6 pg/mL in the non-NIRAF arm, but the difference in PTH levels was not statistically significant (*p* = 0.07). The follow-up period for this study was extended up to six months.

Dip et al. [14] conducted a study in Argentina that included cases of total thyroidectomy performed by experienced surgeons. In their cohort, the overall malignancy rate was 48.8%, while 51.2% of patients were benign. The study focused on NIRAF assessment without ICG angiography, using Fluobeam 800 (Fluoptics, France) for evaluation. Interestingly, they found no statistically significant difference in the mean parathyroid glands identified between the NIRAF arm (3.7) and the non-NIRAF arm (3.6) (*p* = 0.32). The parathyroid gland auto-transplantation rate in the NIRAF arm was 4.7%. Their study defined hypocalcemia as serum calcium levels < 8 mg/dL. The follow-up period for this study was extended up to six months.

Huang et al. [20] conducted a study in China, including cases of total thyroidectomy with or without neck dissection performed by experienced surgeons. Their cohort comprised exclusively of cT1-3, N0-1 papillary carcinoma of the thyroid. The study focused solely on NIRAF assessment without ICG angiography, utilising a handheld infrared camera (Jinan microsmart intelligence technologies, Shandong, China) for evaluation. Significantly, they found a difference in mean parathyroid gland identification between the NIRAF arm (3.9) and the non-NIRAF arm (3.2) (*p* < 0.001). The rate of parathyroid auto-transplantation in the NIRAF arm was significantly lower (6%) compared to the non-NIRAF arm (34%) (*p* < 0.001). The study defined hypocalcemia as serum calcium levels < 8.8 mg/dL. Notably, the mean PTH levels on POD 1 were 21.7 pg/mL in the NIRAF arm and 11.4 pg/mL in the non-NIRAF arm, with the difference in PTH levels being statistically significant (*p* < 0.001). The follow-up period for this study was extended up to six months.

Rossi et al. [8] conducted a study in Italy, including cases of total thyroidectomy with or without neck dissection performed by experienced surgeons. In their cohort, the overall malignancy rate was 58%, with 42% of cases being benign. Their study initially identified the parathyroid glands using NIRAF and assessed the vascularity of the parathyroid gland using ICG angiography, scoring using ICG score following thyroidectomy. For assessment, they utilised Fluobeam LX (Fluoptics, France). The study found a statistically significant difference in mean parathyroid glands identified between the NIRAF arm (3.83) and the non-NIRAF arm (3.64) (*p* = 0.028). Remarkably, the rate of accidental parathyroidectomy was 0% in the NIRAF arm and 4% in the non-NIRAF arm. Furthermore, the rate of parathyroid auto-transplantation was 9% in the NIRAF arm and 34% in the non-NIRAF arm. The study defined hypocalcemia as corrected calcium levels < 8 mg/dL. On POD 1, the mean PTH levels were 16.67 pg/mL in the NIRAF arm and 15.04 pg/mL in the non-NIRAF arm, with the difference in PTH levels not being statistically significant (*p* = 0.165). The follow-up period for this study was extended up to six months.

Lykke et al. [19] conducted a study in Denmark, including cases of total thyroidectomy and completion thyroidectomy performed by experienced surgeons. However, the data from total thyroidectomies only were included in their study. In their cohort, the overall malignancy rate was 59.8%, with 40.2% of cases being benign. Their study identified the parathyroid glands using NIRAF only without ICG angiography, employing Fluobeam 800 (Fluoptics, France) and Elevision IR (Medtronic, Minneapolis, MN, USA) for assessment. Interestingly, the rate of parathyroid auto-transplantation in the NIRAF arm (7.2%) was slightly less than in the non-NIRAF arm (9%) (*p* = 0.94). Their study defined hypocalcemia as ionic calcium levels < 4.72 mg/dL. The follow-up period for this study was extended up to three months.

Wolf et al. [18] conducted a study in Germany, including cases of total thyroidectomy for benign cases performed by experienced surgeons. Their study focused on identifying the parathyroid glands using NIRAF only without ICG angiography, utilising the autofluorescence mode of the Karl Storz endoscopic system with a 3-chip camera for assessment. Interestingly, they found no statistically significant difference in the mean parathyroid glands identified between the NIRAF arm (3.03) and the non-NIRAF arm (3.03) (*p* = 1). The number of parathyroid auto-transplantations in the NIRAF arm was 0.3, while in the non-NIRAF arm, it was 0.4 (*p* = 0.53). The study defined hypocalcemia as calcium levels < 8 mg/dL. On POD 1, the mean PTH levels were 26.7 pg/mL in the NIRAF arm and 24.7 pg/mL in the non-NIRAF arm. Notably, the follow-up period for this study was extended only up to discharge.

Yin et al. [9] conducted a study in China, including cases of total thyroidectomy with neck dissection performed by experienced surgeons for papillary thyroid cancer. Their study initially identified the parathyroid glands using NIRAF and assessed the vascularity of the parathyroid gland using ICG angiography, scoring the vascularity using ICG score. They used an infrared camera from Nanjing Nouyuan Medical Devices, China, for assessment. The study revealed a statistically significant difference in the mean parathyroid glands identified between the NIRAF arm (3.6) and the non-NIRAF arm (3.2) (*p* < 0.001). The number of parathyroid auto-transplantations in the NIRAF arm was 2.3; in the non-NIRAF arm, it was 2.2 (*p* = 0.6). Hypoparathyroidism was defined as PTH < 12 pg/dL in their study. On POD 1, the mean PTH levels were 21.1 pg/mL in the NIRAF arm and 15 pg/mL in the non-NIRAF arm, with the difference in PTH levels being statistically significant (*p* < 0.001).

Papavramidis et al. [17] conducted a study in Greece, including cases of total thyroidectomy performed by experienced surgeons with the ultrasonic scalpel. In their cohort, the overall malignancy rate was 49.4%, while 50.6% of cases were benign. Their study focused on identifying the parathyroid glands using NIRAF only without ICG angiography, employing Fluobeam LX (Fluoptics, France) for assessment. Notably, all four parathyroid glands were found in 29% of cases, and three were identified in 24.2% in the NIRAF arm. However, data for the non-NIRAF arm were unavailable, as the parathyroid glands in the non-NIRAF arm were not searched during surgery. The study found a rate of accidental parathyroidectomy at 14.4% in the NIRAF arm and 28.9% in the non-NIRAF arm (*p* = 0.02). Their study defined hypocalcemia as serum calcium levels < 8 mg/dL. On POD 1, the mean PTH levels were 30.4 pg/mL in the NIRAF arm and 23.5 pg/mL in the non-NIRAF arm, with the difference in PTH levels being statistically significant (*p* = 0.05). There was no long-term follow-up in their study.

Parfentiev et al. [16] conducted a study in Ukraine, including cases of total thyroidectomy performed by experienced surgeons. Their study initially identified the parathyroid glands using NIRAF and assessed the vascularity of the parathyroid gland using ICG angiography; the autofluorescence mode of the Karl Storz endoscopic system with a 3-chip camera was used for assessment. In their cohort, the overall malignancy rate was 18.9%, with 81.1% of cases being benign. Their study defined hypocalcemia as serum calcium levels < 8 mg/dL. On POD 1, the mean PTH levels were 30.08 pg/mL in the NIRAF arm and 30.17 pg/mL in the non-NIRAF arm, with the difference in PTH levels not being statistically significant (*p* = 0.52). Their study followed the participants for a period of up to six months.

The present meta-analysis demonstrates that the use of NIRAF led to a statistically significant difference in the overall rate of hypocalcemia log(OR) = −0.7 [(−1.01, −0.40), M-H, REM, CI = 95%] and temporary hypocalcemia log(OR) = −0.8 [(−1.01, −0.59), M-H, REM, CI = 95%]. The difference in the rate of permanent hypocalcemia log(OR) = −1.09 [(−2.34, 0.17), M-H, REM, CI = 95%] between the two arms trended towards the NIRAF arm. The lack of significance in permanent hypocalcemia rates can be attributed to the small effect size in that group. The included randomised studies had low heterogeneity due to similar inclusion and exclusion criteria (Supplementary Figure S2).

A meta-analysis conducted by Diego Barbieri and colleagues [35] yielded similar outcomes, although their analysis included non-randomised studies, which increased heterogeneity in their groups. The systematic review by Wei Lu et al. [36] and Wang et al. [37] demonstrated that NIRAF reduces inadvertent parathyroid gland excision during total thyroidectomy.

Considering our results, it is reasonable to assume that parathyroid glands sustain less damage during surgery performed with NIRAF than without NIRAF, resulting in higher serum calcium levels in the immediate postoperative period for patients in the NIRAF group. The use of NIRAF during thyroid surgery has been shown to increase the number of glands identified [38] and has been shown to increase the rate of auto-transplanted parathyroid glands, presumably due to improved detection on the specimen. NIRAF can be utilised multiple times throughout the procedure. NIRAF can also potentially replace the frozen section examination of parathyroid glands before auto-transplantation, reducing surgical time and enabling more available tissue for reimplantation [39].

Despite the reported advantages, the adoption of NIRAF has some limitations. NIRAF can be influenced by adipose tissue surrounding parathyroid glands, reducing fluorescence and intraoperative visualisation, as it cannot penetrate beyond 3 mm [40]. Moreover, this technology cannot differentiate between vascularised and devascularised parathyroid glands, and NIRAF does not give information on parathyroid gland viability [39]. The additional use of the intravenous contrast agent ICG can help determine viability [41]. Additionally, the timing of PTH measurement also plays a significant role in determining postoperative hypocalcemia, and there is no standardised timing for measuring PTH following NIRAF use [42].

The introduction of these new technologies inevitably leads to longer operative times. A study by Lerchenberger et al. [43] comparing NIRAF during thyroid surgery showed that its use increased the duration of the operation by approximately 10 min. However, considering the avoidance of postoperative hypocalcemia with NIRAF, this additional time can be deemed a small trade-off.

Our meta-analysis has some limitations. Firstly, the surgeries were performed by experienced surgeons, which may limit the general applicability of the findings. Including surgeons with varying experience levels would have been beneficial to provide a more comprehensive understanding of the results. Given that NIRAF provides an added dynamic in parathyroid gland identification, we feel it may accelerate learning curves for surgical trainees. Additionally, important factors that could influence parathyroid gland preservation, such as malignancy, thyroid gland size, extrathyroidal extension, central compartment clearance, and revision surgery, were not assessed, potentially affecting the conclusions. Especially more extensive surgery puts parathyroid glands and their vascular supply at increased risk and may affect postoperative hypocalcemia rates. Moreover, incorporating NIRAF into the surgical techniques showed slight variations across the included studies. It is also possible to detect NIRAF with a probe-based system (PTEye) [44]. But none of the RCTs have utilised the PTEye method. The effect size calculation and meta-analysis could not be conducted for the number of parathyroid gland identifications, accidental parathyroidectomies, parathyroid auto-transplantation rate, and mean POD 1 PTH levels. It is essential to have a standard deviation, variance, and standard error data to calculate effect sizes using the generic inverse variance method for continuous variables. But these variables were not reported.

Furthermore, another limitation arises from the varying definitions of hypocalcemia used across some studies. Some studies utilised serum calcium, while others used corrected calcium levels. Lastly, the cost-effectiveness of the NIRAF technique needs further assessment to determine its practical value and potential implications in clinical settings.

## 6. Conclusions

NIRAF imaging in thyroid surgery has shown significant benefits by reducing overall and temporary hypocalcemia rates, with a tendency to favour NIRAF for permanent hypocalcemia rates. NIRAF allows for the identification and preservation of parathyroid glands. However, limitations exist, such as the influence of surrounding adipose tissue and the inability to differentiate between vascularised and devascularised glands. Nevertheless, NIRAF holds promise as a valuable tool in thyroid surgery. Further research and cost-effectiveness studies are required for routine adoption.

## Figures and Tables

**Table 1:** Summary studies included for meta-analysis.

Author	Country	Year	Study Type	LOE	NOS	n	NIRAF Arm	No-NIRAF Arm	Overall Hypocalcemia				Temporary Hypocalcemia				Permanent Hypocalcemia			
									NIRAF		No-NIRAF		NIRAF		No-NIRAF		NIRAF		No-NIRAF	
									Yes	No	Yes	No	Yes	No	Yes	No	Yes	No	Yes	No
Dip et al. [14]	Argentina	2019	RCT	1b	9	170	85	85	7	78	14	71	7	78	14	71	0	85	0	85
Benmiloud et al. [15]	France	2020	RCT	1b	9	245	121	120					11	110	26	94	0	121	2	118
Parfentiev et al. [16]	Ukraine	2021	RCT	2b	8	58	30	28	2	28	5	23	2	28	5	23	0	30	1	27
Papavramidis et al. [17]	Greece	2021	RCT	2b	8	180	90	90	3	87	5	85	3	87	5	85				
Yin et al. [9]	China	2022	RCT	2b	8	180	90	90	25	65	39	51	25	65	39	51	0	90	0	90
Wolf et al. [18]	Germany	2022	RCT	2b	8	60	30	30	7	23	14	16	7	23	14	16				
Lykke et al. [19]	Denmark	2023	RCT	1b	9	170	40	44	18	22	21	23	4	36	6	38				
Rossi et al. [8]	Italy	2023	RCT	1b	9	200	100	100					12	88	18	82	1	99	4	96
Huang et al. [20]	China	2023	RCT	2b	8	100	50	50	18	32	30	20	17	33	31	19	0	50	1	49
Overall		1363					636	637	80	335	128	289	88	548	158	479	1	475	8	465

RCT—Randomised control trial; LOE—Level of evidence; NOS—Newcastle–Ottawa scale; NIRAF—Near Infrared autofluorescence.

**Table 2:** Detailed description of included studies. Summary of included studies.

No.	Cohort	Malignancy vs. Benign	Type of Assessment	NIRAF Equipment	Parathyroid Gland Identification	Accidental Parathyroidectomy	Parathyroid Auto-Transplantation Rate	Definition of Hypocalcemia	Mean POD 1 PTH Levels
1	Dip et al. [14]	Malignant: 48.8%; benign cases: 51.2%	NIRAF only	Fluobeam 800 (Fluoptics, France)	NIRAF (3.7) vs. non-NIRAF arm (3.6) (*p* = 0.32)		NIRAF arm: 4.7%	Serum calcium levels < 8 mg/dL	
2	Benmiloud et al. [15]	Malignant: 23.2%; benign cases: 76.8%	NIRAF only	Fluobeam 800 (Fluoptics, France)	All 4-gland identification in NIRAF (47%) vs. non-NIRAF arm (19.2%) (*p* < 0.001)	NIRAF arm (2.4%) vs. non-NIRAF arm (11.5%) (*p* = 0.006)	NIRAF arm: 2.4%; non-NIRAF arm: 11.5% (*p* = 0.006)	Corrected calcium levels < 8 mg/dL	NIRAF arm: 33.2 pg/mL; non-NIRAF arm: 28.6 pg/mL (*p* = 0.07)
3	Papavramidis et al. [17]	Malignant: 49.4%; benign cases: 50.6%	NIRAF only	Fluobeam LX (Fluoptics, France)	All 4 parathyroid glands were found in 29% of cases, and 3 glands were identified in 24.2% of the NIRAF arm	NIRAF arm (14.4%) vs. non-NIRAF arm (28.9%) (*p* = 0.002)		Hypocalcemia defined as serum calcium levels < 8 mg/dL	NIRAF arm: 30.4 pg/mL; non-NIRAF arm: 23.5 pg/mL (*p* = 0.005)
4	Parfentiev et al. [16]	Malignant: 18.9%; benign cases: 81.1%	NIRAF followed by ICG angiography	Autofluorescence mode of Karl Storz endoscopic system with 3 chip cameras				Hypocalcemia defined as serum calcium levels < 8 mg/dL	NIRAF arm: 30.08 pg/mL; non-NIRAF arm: 30.17 pg/mL (*p* = 0.52)
5	Wolf et al. [18]	Benign: 100%	NIRAF only	Autofluorescence mode of Karl Storz endoscopic system with 3 chip cameras	NIRAF (3.03) vs. non-NIRAF arm (3.03) (*p* = 1)		NIRAF arm: 0.3; non-NIRAF arm: 0.4 (*p* = 0.53)	Hypocalcemia defined as serum calcium levels < 8 mg/dL	NIRAF arm: 26.7 pg/mL; non-NIRAF arm: 24.7 pg/ml
6	Yin et al. [9]	Malignant: 100%	NIRAF followed by ICG angiography	Infrared camera (Nanjing Nouyuan Medical device, China)	NIRAF (3.6) vs. non-NIRAF arm (3.2) (*p* < 0.001)		NIRAF arm: 2.3; non-NIRAF arm: 2.2 (*p* = 0.6)	*Hypoparathyroidism = PTH < 12 pg/mL	NIRAF arm: 21.1 pg/mL; non-NIRAF arm: 15 pg/mL (*p* < 0.001)
7	Huang et al. [20]	Malignant: 100%	NIRAF only	Infrared camera (Jinan microsmart intelligence technologies, China)	NIRAF (3.9) vs. non-NIRAF arm (3.2) (*p* < 0.001)		NIRAF arm: 6%; non-NIRAF arm: 34% (*p* < 0.001)	Serum calcium levels < 8.8 mg/dL	NIRAF arm: 21.7 pg/mL; non-NIRAF arm: 11.4 pg/mL (*p* < 0.001)
8	Rossi et al. [8]	Malignant: 58%; benign cases: 42%	NIRAF followed by ICG angiography	Fluobeam LX (Fluoptics, France)	NIRAF (3.83) vs. non-NIRAF arm (3.64) (*p* = 0.028)	NIRAF arm (0%) vs. non-NIRAF arm (4%)	NIRAF arm: 9%; non-NIRAF arm: 34%	Corrected calcium levels < 8 mg/dL	NIRAF arm: 16.67 pg/mL; non-NIRAF arm: 15.04 pg/mL (*p* = 0.165)
9	Lykke et al. [19]	Malignant: 59.8%; benign cases: 40.2%	NIRAF only	Fluobeam 800 (Fluoptics, France) and Elevision IR (Medtronic, USA)			NIRAF (7.2%) vs. non-NIRAF arm (9%) (*p* = 0.94)	Ionic calcium levels < 4.72 mg/dL	

**Table 3:** Overview of meta-analysis statistics.

	Overall Hypocalcemia	Temporary Hypocalcemia	Permanent Hypocalcemia
Number of studies (k)	k = 7	k = 9	k = 6
Log odds ratios	Range: −1.11 to −0.11	Range: −1.15 to −0.35	Range: −1.63 to 0
Majority of estimates	Negative (100%)	Negative (100%)	Negative (in 4 out of 6 studies, 67%)
Estimated average log odds ratio	\hat{\μ} = −0.7	\hat{\μ} = −0.8	\hat{\μ} = −1.08
95% confidence interval	−1.05 to −0.35	−1.11 to −0.49	−2.34 to 0.17
Significance of average outcome	z-score = −3.95, *p*-value < 0.001	z-score = −5.1375, *p*-value < 0.001	z-score = −1.6953, *p*-value = 0.09
Maximum weightage	Yin et al. [9] (31.6%)	Yin et al. [9] (24.2%)	Rossi et al. [8] (32.3%)
Heterogeneity	Q-test *p*-value = 0.81, tau^2^ = 0, I^2^ = 0%	Q-test *p*-value = 0.95, tau^2^ = 0, I^2^ = 0%	Q-test *p*-value = 0.98, tau^2^ = 0, I^2^ = 0%
Outliers	No outlier studies, Studentised residuals < ±2.69	No outlier studies, Studentised residuals < ±2.77	No outlier studies, Studentised residuals < ±2.64
Influential studies	None, Cook’s distances < 1	None, Cook’s distances < 1	None, Cook’s distances < 1
Funnel plot asymmetry	Rank correlation *p*-value = 1, regression test *p*-value = 0.73	Rank correlation *p*-value = 1, regression test *p*-value = 0.98	Rank correlation *p*-value = 0.14, regression test *p*-value = 0.49

## Data Availability

All data generated or analysed during this study are included in this article. Further enquiries can be directed to the corresponding author.

## Supplementary Materials

The following supporting information can be downloaded at: https://www.mdpi.com/article/10.3390/diagnostics14050505/s1, Figure S1: Risk of bias A. Graph, B. Summary; Figure S2: A: Funnel plot for overall rate of postoperative hypocalcemia. B: Funnel plot for the rate of temporary hypocalcemia. C: Funnel plot for the rate of permanent hypocalcemia. D: Radial plot for overall rate of postoperative hypocalcemia. E: Radial plot for the rate of temporary hypocalcemia. F: Radial plot for the rate of permanent hypocalcemia.
