# Supplementary material for: Lower Rates of Hypocalcemia Following Near-Infrared Autofluorescence Use in Thyroidectomy: A Meta-Analysis of RCTs
Source: Diagnostics (Basel). 2024 Feb 27;14(5):505. doi: 10.3390/diagnostics14050505 (PMC10931323; doi:10.3390/diagnostics14050505)
Supplement: Supplementary file 1 [file diagnostics-14-00505-s001.zip › diagnostics-2808495-supplementary.pdf]

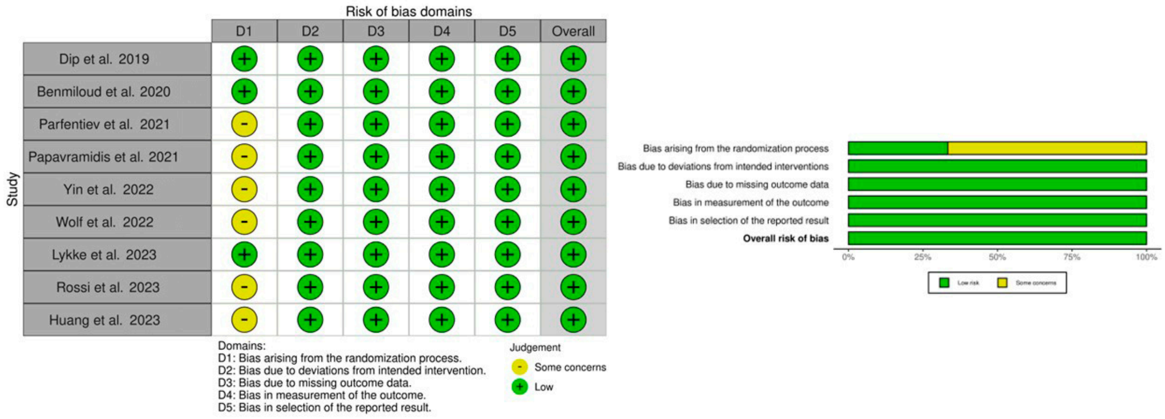

Supplemental Figure S1. Risk of bias A. Graph, B. Summary [8,9,14–20].

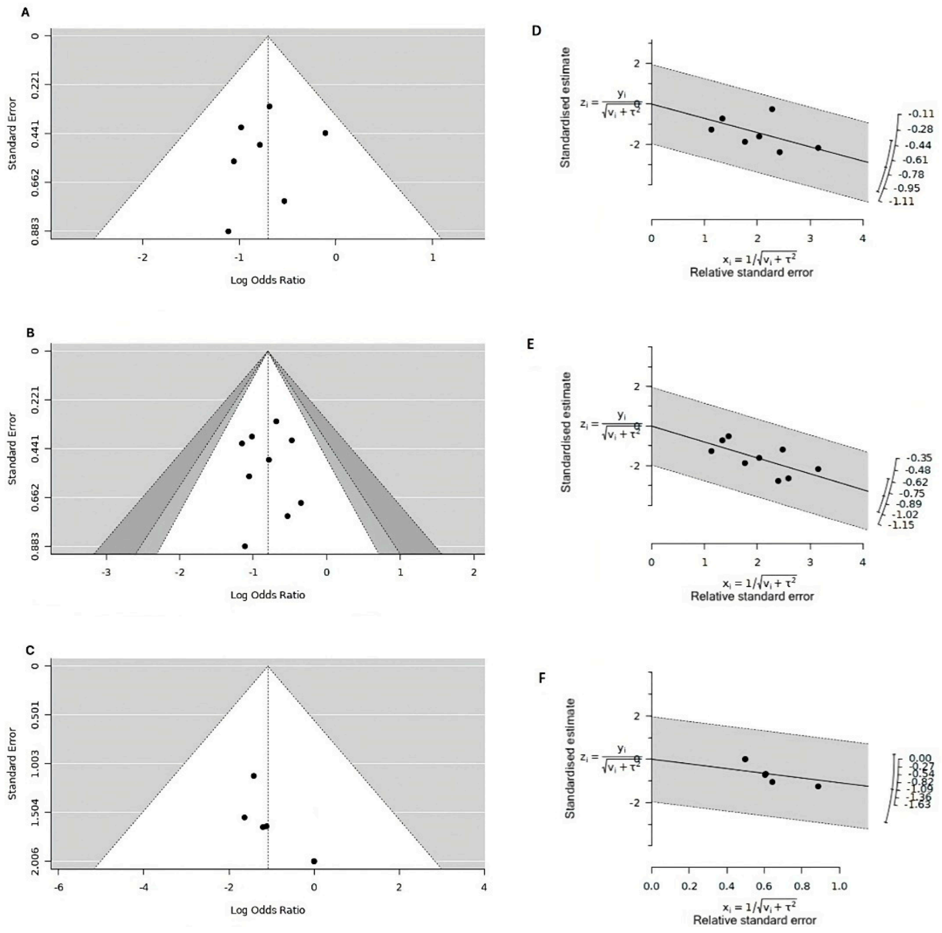

Supplemental Figure S2. A: Funnel plot for overall rate of postoperative hypocalcemia. B: Funnel plot for the rate of temporary hypocalcemia. C: Funnel plot for the rate of permanent hypocalcemia. D: Radial plot for overall rate of postoperative hypocalcemia. E: Radial plot for the rate of temporary hypocalcemia. F: Radial plot for the rate of permanent hypocalcemia.
